# Novel bronchoscopic balloon dilation for patients with bronchostenosis caused by bronchial tuberculosis: a case report

**DOI:** 10.1186/1752-1947-8-225

**Published:** 2014-06-24

**Authors:** En-Qing Fu, Fa-Guang Jin

**Affiliations:** 1 Department of Respiratory and Critical Medicine of Tangdu Hospital, the Fourth Military Medical University, Xi’an, China

**Keywords:** Balloon dilation, Bronchostenosis, Video-bronchoscopy

## Abstract

**Introduction:**

Bronchoscopic balloon dilation is a common method in the treatment of bronchostenosis but it is not an effective treatment due to its short dilating time (3 minutes) and low pressure (<3atm). Until recently, the reported highest dilating pressure was ≤6atm; however, this is not enough pressure to dilate a bronchostenosis because of the resistance of the bronchus. We hypothesized that higher dilating pressure (up to 14atm) with longer dilating time (40 minutes) may make bronchoscopic balloon dilation treatment more effective according to the blood vessel dilating method. Therefore, we designed this new bronchoscopic balloon dilation method for treating bronchostenosis, particularly in cases caused by bronchial tuberculosis.

**Case presentation:**

A 23-year-old Chinese woman presented with right middle segmental bronchostenosis caused by bronchial tuberculosis. She was informed of the surgical procedure and she provided informed consent. After taking anti-bronchial tuberculosis drugs for 2 months, she underwent our new bronchoscopic balloon dilation treatment (dilating time, 40 minutes; pressure, 14atm). After anti-bronchial tuberculosis treatment for 13 months, her intermediate bronchus was observed with videobronchoscopy again and no re-stenosis was seen. Furthermore, a computed tomography scan revealed that her right lower lobe and right middle lobe had reopened. No complications occurred in the patient.

**Conclusion:**

The novel high-handed videobronchoscopic balloon dilation method was safe and effective for treating this patient with bronchostenosis caused by bronchial tuberculosis.

## Introduction

Bronchoscopic balloon dilation (BBD) is commonly used in the treatment of bronchostenosis. However, it is not effective in treating innocent bronchostenosis due to its short dilating time (3 minutes) and low pressure (<3atm) [1,2]. We designed a new BBD method for treating benign bronchostenosis caused by bronchial tuberculosis (TB) which utilizes higher dilating pressure (up to 14atm) and longer dilating time (40 minutes) according to the blood vessel dilating method. The patient described in this case report was informed of the whole procedure prior to her operation and she provided informed consent.

## Case presentation

**Figure 1:**
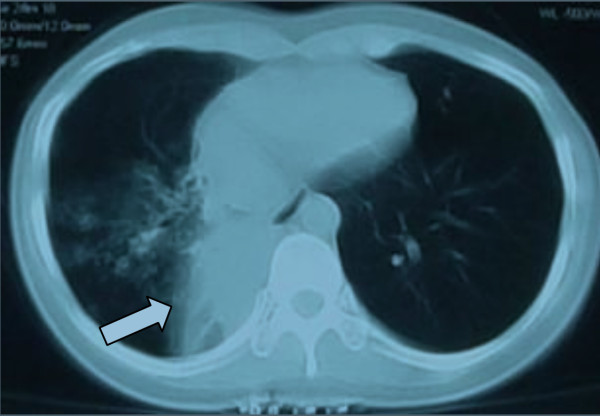
**Computed tomography scan of the chest showed partial atelectasis of the right lower lobe (arrow) before treatment.** (13 October 2011).

**Figure 2:**
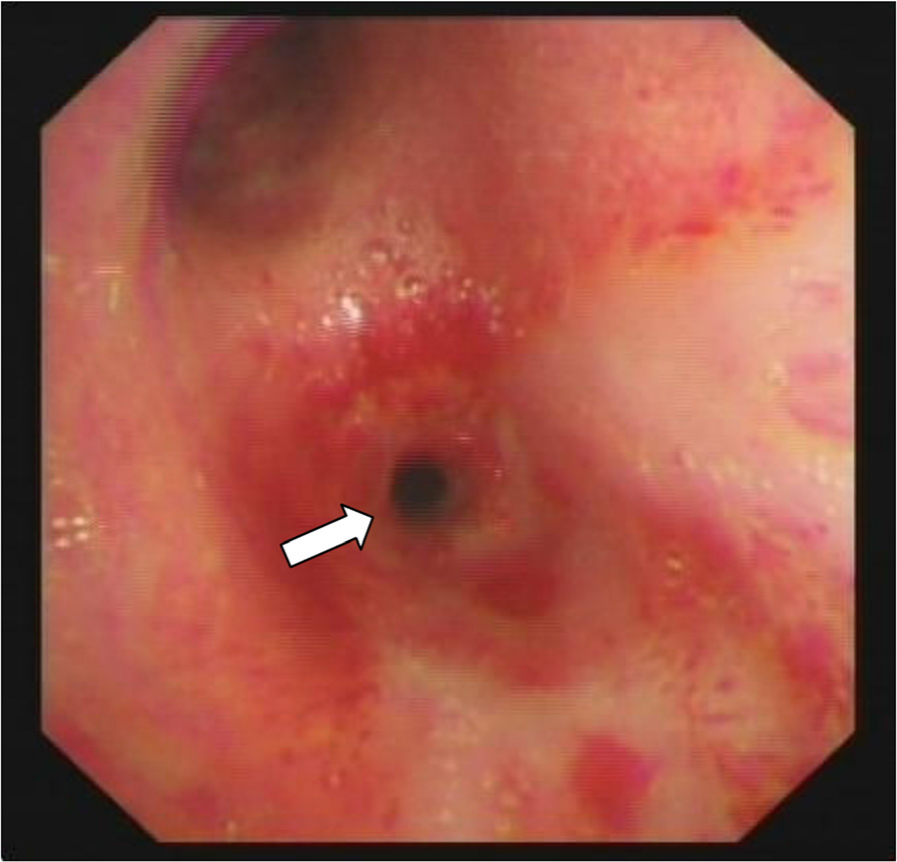
**The intermedial segment bronchus was in severe stenosis with a diameter of approximately 3mm before dilation (arrow).** (2 November 2011).

**Figure 3:**
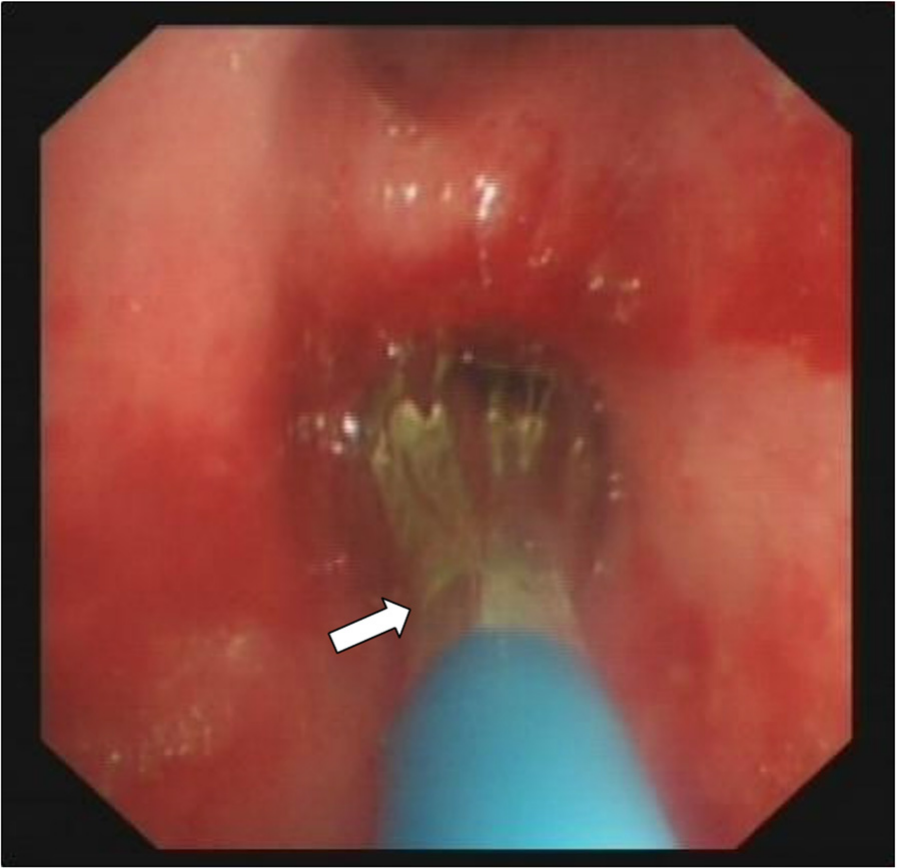
**The balloon was at the narrow part of the intermedial segment bronchus (arrow).** (2 November 2011)

**Figure 4:**
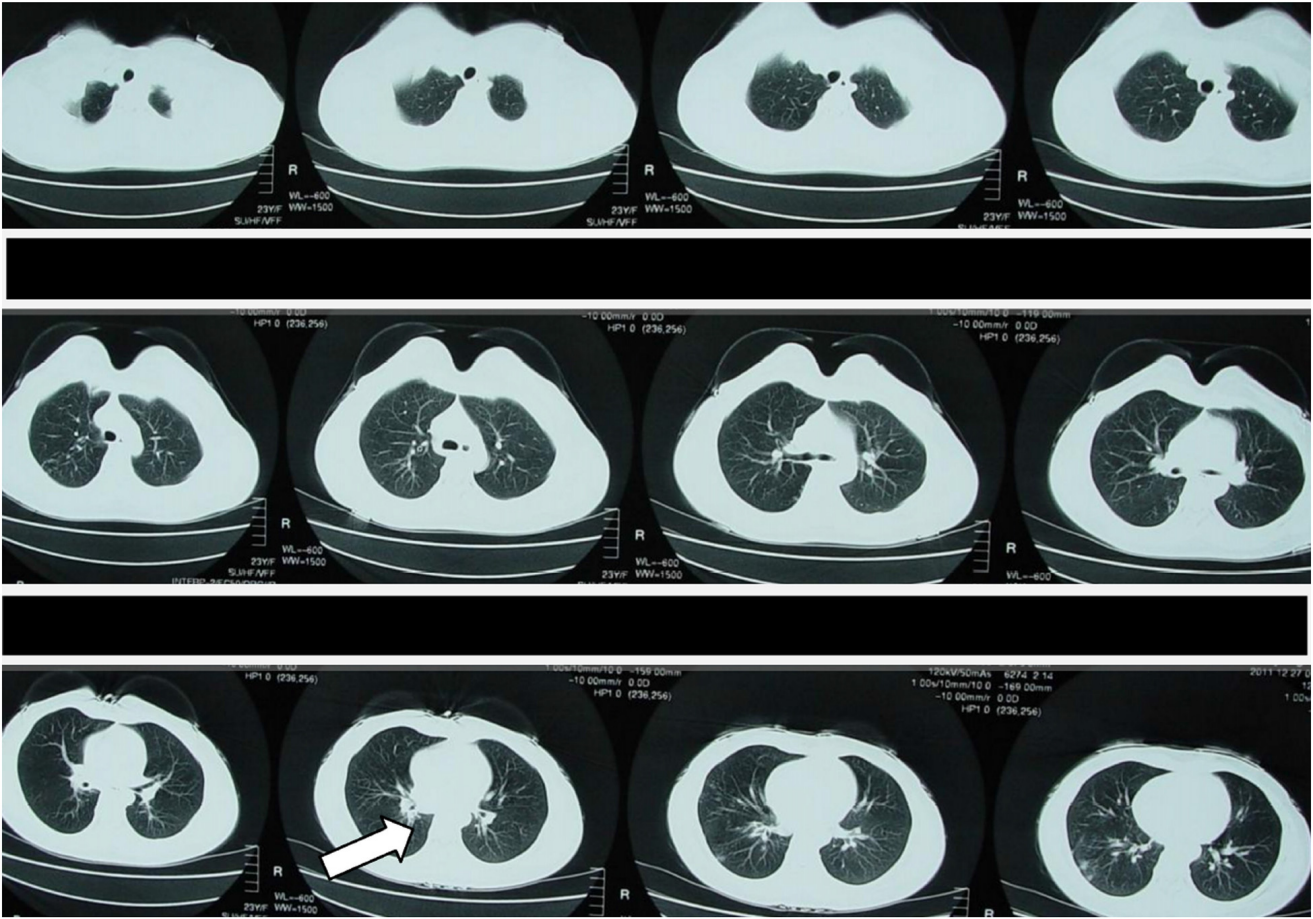
**The right lower lobe was gradually stretched to fully open after dilation (arrow).** (27 December 2011)

**Figure 5:**
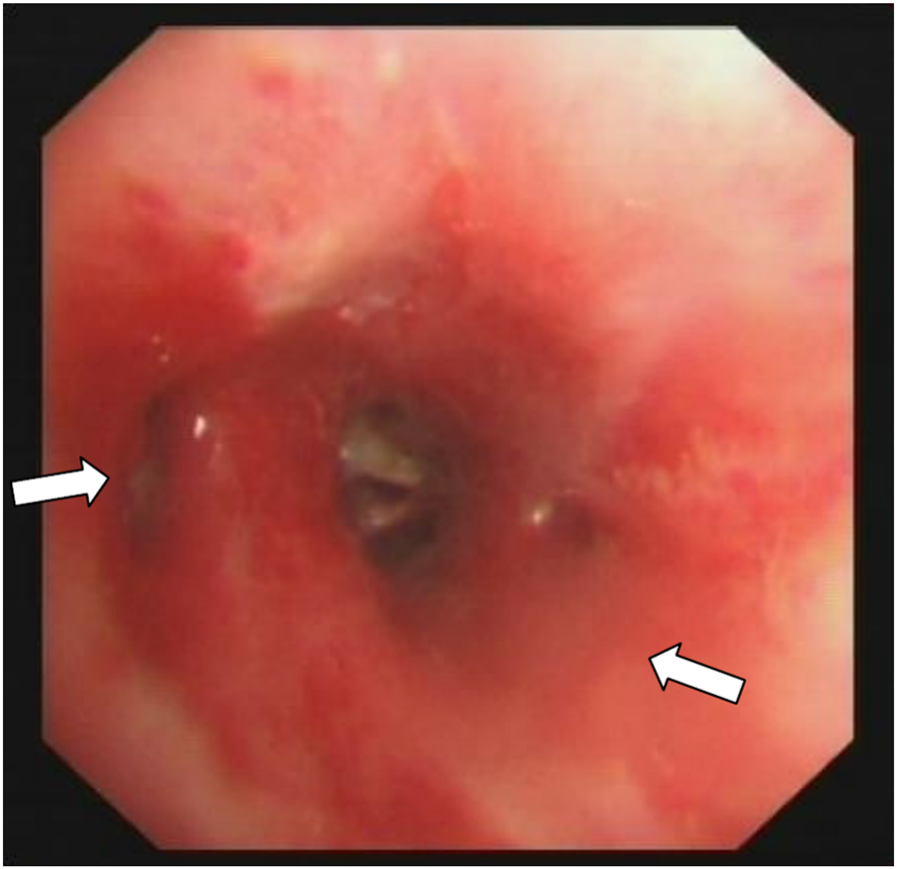
**The middle and lower lobe of the bronchus were recovered after balloon dilation (arrows).** (27 January 2012).

A 23-year-old Chinese woman presented with cough, expectoration, wheeze and dyspnea for 3 months. A physical examination indicated weakened respiratory sounds in her right lower lobe. A chest computed tomography (CT; Figure 1) showed partial atelectasis of her right lower lobe. Her intermedial segment bronchus was in severe stenosis, with a diameter of approximately 3mm (Figure 2). Our final diagnosis was pulmonary TB combined with bronchial TB based on the detection of tubercle bacilli from her sputum. She took isoniazid (0.3g/day), rifampicin (0.45g/day), ethambutol (0.75g/day) and pyrazinamide (0.5 three times daily) for 2 months.After attaining her signed informed consent and the permission of the Ethics Committee of our hospital, we performed a high-pressure balloon dilation in her intermediate bronchus through videobronchoscope (BF-260, Olympus Corp., Tokyo, Japan), with the balloon produced by Johnson & Johnson Corporation of USA in China, 10mm diameter ×40mm length (Figure 3).The procedure for our high-handed videobronchoscopy balloon dilation was as follows: A) Insert the bronchoscope into her bronchus. B) Insert the guide wire along the working hole of the bronchoscope. C) Pull out the videobronchoscope from her bronchus and reinsert the bronchoscope into her bronchus. D) Insert the high-handed balloon along the guide wire into the narrowest part of her bronchus and inject physiologic saline through the balloon duct with a booster pump to increase the pressure of the balloon (from 0 to 14atm). E) Remove the bronchoscope from her bronchus. She was seated for 40 minutes with continual balloon dilation at high pressure (14atm) and without the videobronchoscope in her bronchus. F) Re-insert the bronchoscope into her bronchus and remove the balloon and the guide wire from her bronchus. G) Remove the bronchoscope from her bronchus after the operation is finished. One month after continuous anti-TB treatment, CT film showed that gradual expansion of her right lower lobe had occurred until it had completely opened (Figure 4), and her clinical symptoms had disappeared. An examination using an electronic bronchoscope showed the diameter of the intermediate bronchus was 9mm (Figure 5). Five weeks later, she was checked twice through the bronchoscope and there was no re-stenosis in her intermediate bronchus. She then took anti-TB drugs for 12 months and recovered completely and without re-stenosis. No re-stenosis was observed after continuous anti-TB treatment for 13 months. There were no side effects.

## Discussion

Balloon dilation is an alternative method for treating patients with bronchostenosis. However, traditional methods of BBD just insert the catheter-balloon into the work hole of the bronchoscope and directly dilate the bronchostenosis. Carlin *et al.*[3] used a catheter-balloon to dilate bronchostenosis through a rigid bronchoscope. Their method of dilation was 4 to 5 times 1 round which was just inflated once for 30 seconds and the dilating pressure was below 6atm so it did not achieve a satisfactory effect due to its short duration and low dilating pressure. Shitrit *et al.*[1] and Garg *et. al*[4] used a 7-Fr, 10mm angioplastic balloon catheter (Medi-tech, Watertown, MA, USA) to dilate bronchostenosis for 10 seconds, with a maximal pressure of 6atm (6.1 to 101Pa) through a fibrobronchoscope. Although the method was effective and safe, its effect failed to last long and re-stenosis of bronchus occurred several days later due to its lower pressure and short duration of dilation. We designed novel balloon dilation with high pressure (above 10atm) and a duration (>10 minutes) longer than is normally used in blood vessel dilation for patients with vessel stenosis. Our balloon dilation has confirmed that 40 minutes was long enough for bronchostenosis to be reopened without causing mucous membrane necrosis and bronchus slit. Compared with other methods [5] of balloon dilation in the treatment of patients with bronchostenosis, the high pressure of our balloon dilation is 14atm according to the high-handed balloon requirement and the dilating time is determined by the effect of dilation. The traditional BBD is performed through a videobronchoscope work hole and has a videobronchoscope inserted in the bronchus, which makes the patient feel panic. Our balloon dilation decreased the side effects of traditional BBD, such as hemorrhage, lacerations, pneumothorax, mediastinal emphysema and re-stenosis. In this patient, there were no side effects. Therefore, this self-designed novel high-handed videobronchoscopy balloon dilation is reliable and effective in the treatment of benign bronchostenosis but it is not suitable for trachea stenosis treatment.

## Conclusion

This case report revealed that this novel BBD is a more steady effective method to treat bronchostenosis caused by TB compared with the traditional BBD method.

## Consent

Written informed consent was obtained from the patient for the publication of this case report and accompanying photographs and scanned images. A copy of the written consent is available for review by the Editor-in-Chief of this journal.

## Abbreviations

BBD: Bronchoscopic balloon dilation; CT: Computed tomography; TB: Bronchial tuberculosis.

## Competing interests

The authors declare that they have no competing interests in this article.

## Authors’ contributions

EQF collected clinical information, designed and performed the BBD treatment, and wrote the manuscript. FGJ guided BBD treatment and polished the manuscript. Both authors have approved of the publication of the case report.

## Authors’ information

En-Qing Fu (MD), Professor and Vice-Director of Department of Respiratory and Critical Medicine of Tangdu Hospital, the Fourth Military Medical University (Xinsi Roard of Baqiao District, Xi’an , Shaanxi, China, +86-710038), who has been engaged in micro-invasive treatment via videobronchoscopy for more than 10 years.

Fa-Guang Jin (MD) (PHD), Professor and Director of Department of Respiratory and Critical Medicine of Tangdu Hospital, the Fourth Military Medical University (Xinsi Roard of Baqiao District, Xi’an, Shaanxi, China, +86-710038).
